# Expression of Concern: Atta et al. Novel Magnetic Silica-Ionic Liquid Nanocomposites for Wastewater Treatment. *Nanomaterials* 2020, *10*, 71

**DOI:** 10.3390/nano16191230

**Published:** 2026-09-30

**Authors:** Nanomaterials Editorial Office

**Affiliations:** MDPI AG, Grosspeteranlage 5, 4052 Basel, Switzerland; nanomaterials@mdpi.com

With this notice, the *Nanomaterials* Editorial Office alerts readers to concerns regarding this article [1].

Following publication, concerns were raised regarding the accuracy of Figures 4 and 6. In accordance with the journal’s standard procedures, the Editorial Office and Editorial Board assessed the concerns and sought clarification from the authors. However, despite repeated attempts to obtain additional information, the authors could not be reached, and the concerns could not be conclusively resolved.

After evaluating the available evidence, the Editorial Board concluded that the concerns relating to specific figures do not substantially affect the overall findings or conclusions presented in the article.

This Expression of Concern is issued to inform readers of the unresolved concerns regarding Figures 4 and 6, which should therefore be interpreted with appropriate caution. No further editorial action is planned at this time. However, should additional information become available, the Editorial Office and Editorial Board may consider further action in accordance with MDPI’s policies.
